# Supplementary material for: Identification of a PGXPP degron motif in dishevelled and structural basis for its binding to the E3 ligase KLHL12
Source: Open Biol. 2020 Jun 24;10(6):200041. doi: 10.1098/rsob.200041 (PMC7333892; doi:10.1098/rsob.200041)
Supplement: Supplementary Figures 1 and 2 [file rsob200041supp1.pdf]

# Identification of a PGXPP degron motif in dishevelled and structural basis for its binding to the E3 ligase KLHL12

Zhuoyao Chen, Gregory A. Wasney, Sarah Picaud, Panagis Filippakopoulos, Masoud Vedadi, Vincenzo D'Angiolella and Alex N. Bullock

## Supplementary Material:

## Figure S1

## Figure S2

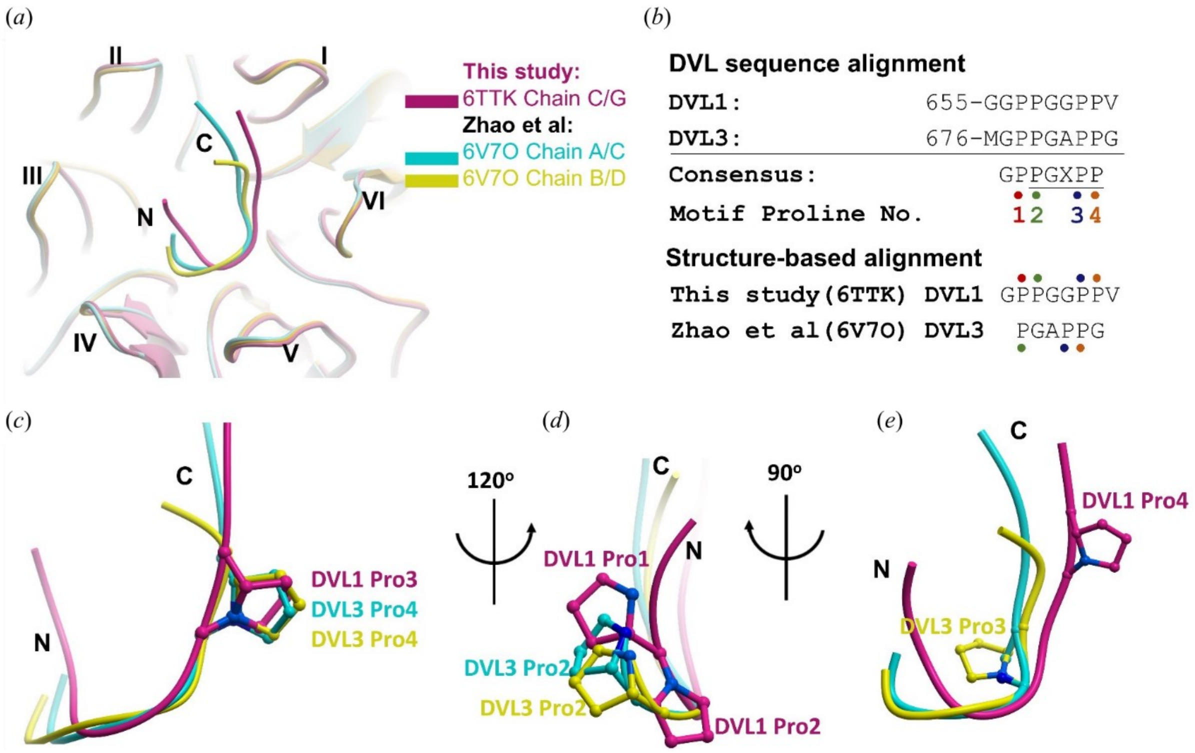


**Figure S1. Structural superposition of 6TTK (this study, KLHL12-DVL1) and 6V7O (Zhao et al, KLHL12-DVL3) reveals a shift in the packing of the bound DVL peptides.** (a) The overall U-shaped conformation of the bound DVL peptides is maintained in the two co-structures. The DVL1 co-structure (6TTK) is coloured in purple. For the DVL3 co-structure (6V7O), chains A/C and chains B/D are coloured in blue and yellow, respectively. (b) Sequence alignments show that the DVL1-3 degron motifs contain four prolines within a conserved PPGXPP motif. For clarity, the prolines in this region are numbered 1 to 4 as indicated and highlighted by red, green, blue and orange spots, respectively. Superposition of the two complexes reveals a different structure-based alignment for the crystallised DVL peptides that likely reflects the fact the first proline position was truncated from the DVL3 peptide crystallised by Zhao et al. The DVL1 co-structure revealed buried interface positions for the first, second and third prolines in this motif, while the fourth proline was largely exposed (Figure 4). By comparison, the truncated DVL3 peptide appears to have induced a shift in the bound peptide position (c-e) such that the conserved motif's second, third and fourth prolines are now buried. (c) Structural superposition of the two KLHL12 complexes reveals that 'proline 3' in DVL1 aligns well with 'proline 4' from the DVL3 co-structure, suggesting that this proline position is favoured for KLHL12 interaction, as described in Figure 4e. (d) The N-terminal truncation of the DVL3 peptide of Zhao et al also prevents the formation of two intramolecular hydrogen bond interactions that stabilise the bound DVL1 conformation (Figure 3b). This may contribute to the inconsistent N-terminal conformations of DVL3 chains C and D in 6V7O. (e) The DVL1 and DVL3 peptide conformations additionally deviate at their C-termini.

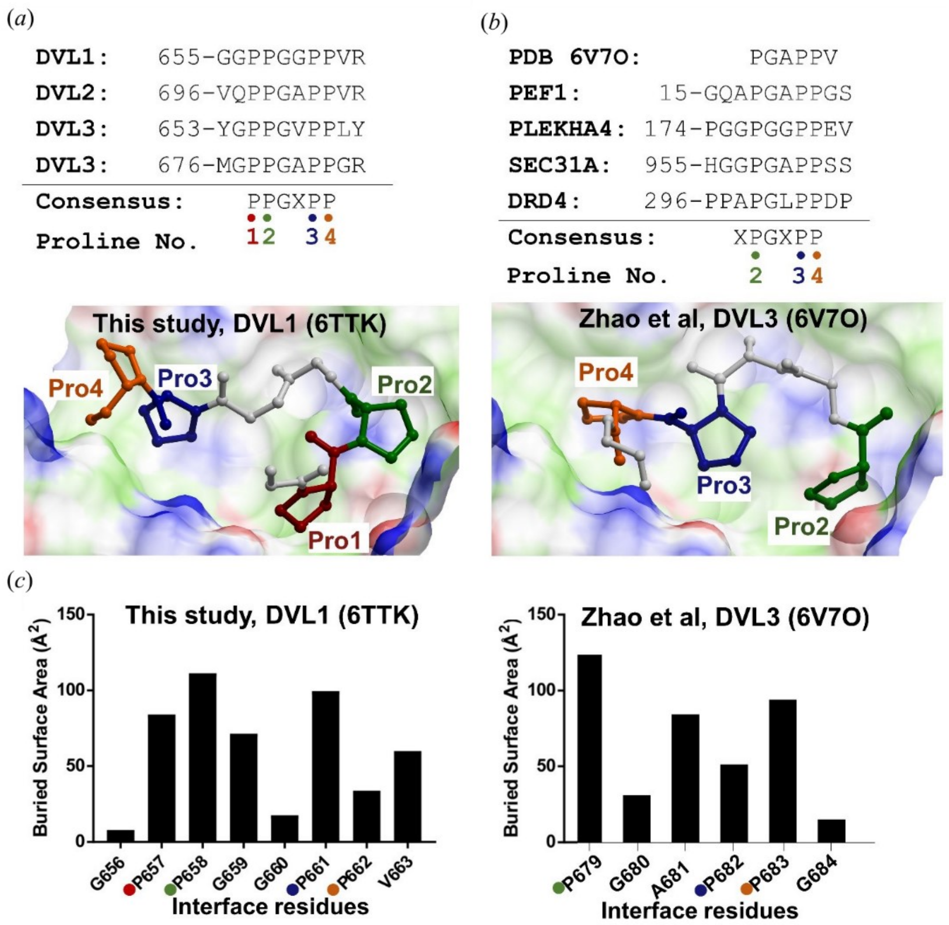


**Figure S2. Structures in this study and in Zhao et al may represent two different binding modes for KLHL12-interacting proteins.** (a) Sequence alignment of DVL1-3 shows a conserved 'PPGXPP' motif at the degron site. The four prolines in region are numbered and colour coded as described in Figure S1. The interface positions of all four prolines are defined in the DVL1 co-structure. Proline residues in DVL1 are coloured to match the sequence alignment. A surface representation is shown for the KLHL12 substrate-binding pocket (blue and red surface positions denote hydrogen bond donor and acceptor sites, respectively). (b) The truncated DVL3 peptide crystallised by Zhao et al omitted the first proline position. DVL3 residues defined in the electron density from this structure are shown in a sequence alignment with other KLHL12-interacting proteins, which also have the first proline position substituted by a glycine or alanine resulting in the shorter consensus motif 'PGXPP'. The structure of Zhao et al suggests that the peptide binding is shifted so that the N-terminal 'proline 2' position can now be accommodated closer to the pocket site otherwise engaged by 'proline 1' in DVL1. We speculate that the DVL1 co-structure may be representative of the dishevelled family binding mode, with their four prolines, whereas the co-structure of Zhao et al may reflect an alternative binding mode for a subgroup of binding partners containing only the C-terminal three prolines. The alternative binding mode likely explains why deletion of the N-terminal proline in DVL1 was well tolerated in the SPOTs peptide array (Figure 2b). (c) Comparison of the buried surface areas of individual DVL residues in the 6TTK (this study) and 6V7O (Zhao et al) structures, respectively.
